# The Underutilization of Adjunctive Pharmacotherapy in Treating Acute Coronary Syndrome Patients Admitted to a Tertiary Care Hospital in Southwest Region, Saudi Arabia

**DOI:** 10.4103/1995-705X.76800

**Published:** 2010

**Authors:** Abdullah S. Assiri

**Affiliations:** King Khalid University, Abha, Saudi Arabia

**Keywords:** Adjunctive, acute coronary syndrome, pharmacotherapy, underutilization

## Abstract

**Background:**

Acute coronary syndrome (ACS) is the most prevalent cardiac disorder. Adjunctive pharmacotherapy has proved to be safe and effective in treating patients with this syndrome. Underutilization of such pharmacotherapy was reported in different studies.

**Objectives:**

In this study, we evaluated the underutilization of these pharmacotherapies on patients admitted to Aseer Central Hospital (ACH) with ACS, find out factors that may predict utilization of these therapies, and determine the effect of such pattern of drug utilization on survival at discharge.

**Materials and Methods:**

A retrospective cohort of 562 patients admitted with the diagnosis of ACS to ACH during the period from March 2007 to February 2009 was studied.

**Results:**

β-blockers (B-blocker) and angiotensin-converting enzyme inhibitors (ACEI) were used in only 69 and 59% of cases, respectively. Aspirin, clopidogrel, and statin were used in 98.4, 82.6, and 89.3% of cases, respectively. The presence of diabetes predicts the use of ACE inhibitors, whereas the diagnosis of unstable angina and ST-elevation myocardial infarction predict the use of statin. Survival rate at discharge was 95.6%. Use of statin and aspirin improved survival.

**Conclusion:**

Certain adjunctive pharmacotherapies were underutilized in ACS patients in Southwest region, Saudi Arabia, specifically β-blockers and ACEI. Standard of care should be revised and updated, aiming to improve adherence to guidelines of management of patients with ACS.

## INTRODUCTION

Acute coronary syndrome (ACS) is the most prevalent cardiac disorder, affecting over 12 million in the United States. Each year, it is estimated that one million individuals in the United States suffer a coronary event.[1]

Despite improvement in primary prevention[2] and treatment,[3] ACS remains the chief cause of death in the United States and most developed countries. Almost half of all victims of myocardial infarction die before they reach the hospital.[4] Of several hundred thousand patients hospitalized each year with ACS, 7 to 15% die during hospitalization and another 7 to 15% die during the following year.[5]

ACS includes ST-segment elevation myocardial infarction (STEMI), non–ST-segment elevation myocardial infarction (NSTEMI), and unstable angina. Adjunctive therapy for ACS includes antiplatelet therapy, beta-adrenergic blocking agents (β-blockers [β-blockers]), angiotensin-converting enzyme inhibitors (ACEI), and lipid-lowering agents (statin).

Several studies have shown survival benefit when these therapies were given to patients with ACS. Anti-platelet therapy (aspirin) is the single most cost-effective adjunctive therapy for ACS treatment. It decreases mortality in treated patients by 23% (ISIS 2).[6]

Multiple controlled trials have demonstrated that β-blocker therapy use for ACS patients decreases both early and late cardiovascular mortality and re-infarction rate, and increases survival by 20 to 40%.[7–11]

The use of ACEI in treating ACS patients reduces mortality post-myocardial infarction by 7% in ISIS-4 trial[12] and by 12% in GISSI-3 trial.[13]

The use of lipid-lowering therapy (statin) in ACS patients has revealed decreased rate of progression and modest regression of atheromatous disease in treated patients. It reduces all-cause mortality by 45%.[14]

Several guidelines were established to improve care for ACS patients.[15,16] These guidelines emphasizes the importance of using these pharmacotherapies in managing patients with ACS.

A wide variation of clinical practice regarding prescription of these adjunctive pharmacotherapies is noticed among different physicians and different institutions, despite the availability of clinical guidelines and good evidence-based data to support the use of such agents. Several studies showed that the recommended medications may not always be used when appropriate.[17,18]

The Aseer region (population of 1 200 000) is located in the southwest of Saudi Arabia, covering an area of more than 80 000 km^2^. The region extends from the high mountains of Sarawat (with an altitude of 3,200m above sea level) to the Red Sea. Health Services delivery in the Aseer region is provided by a network of 244 primary health care centers, 16 referral hospitals, and one tertiary hospital, Aseer Central Hospital (ACH). ACH, with 500 beds, is run by the Ministry of Health and the College of Medicine of King Khalid University (KKU), Abha.

This study tried to establish the pattern of utilization of adjunctive pharmacotherapy for patients admitted to ACH with the diagnosis of ACS, find out how close we are to optimal care, determine the different variables that can affect this pattern, and evaluate the effect of this practice on survival at discharge.

## MATERIALS AND METHODS

This study was a retrospective cohort of all patients admitted to ACH with the diagnosis of ACS for the period between March 2007 and February 2009.

Patient’s data were reviewed and tabulated, including demographic data, coronary risk factors, different adjunctive pharmacologic agents used, and mortality records. Data were analyzed using SPSS software package.

Frequency, percentage, mean, standard deviation, and median were used to present the data. Binary logistic regression analysis was used to identify potential factors affecting utilization of different adjunctive pharmacotherapy used in ACS patients, and factors associated with survival of the study cohort ’at 5% level of significance.’

Approval of the local medical-ethical committee was obtained.

## RESULTS

### Demographic profile and clinical variables of the study patients

The present study included 562 patients with the diagnosis of ACS, 407 (72.4%) were men and 155 (27.6%) were women. The age ranged from 26 to 100 years with an average 60.6 ± 13.7 years and a median of 60 years. The majority of cases were Saudis (502, 89.3%). The distribution of diagnosis was unstable angina (UA) (307, 54.6%), ST-elevation myocardial infarction (190, 33.8%), and non–ST-elevation myocardial infarction (65, 11.6%). More than half of the patients (287, 51.1%) were diabetic. More than one-third of the patients (216, 38.4%) were hypertensive. 18.9% (106) were current smokers.

### Adjunctive pharmacotherapy used

The present study included observations on the following adjunctive pharmacotherapies: Anti-platelet therapy (aspirin and clopidogrel), β-blockers, ACEI (or angiotensin 2 receptor blockers for patients intolerant to ACEI), and lipid-lowering therapy (statin).

Aspirin, clopidogrel, and statin therapies were given to the majority of patients (98.4, 82.6, and 89.3%, respectively).

On the other hand, β-blockers and ACEI (or angiotensin 2 receptor blockers) were given to a less proportion of patients (69 and 59%, respectively).

### Potential factors affecting intake of adjunctive pharmacotherapy

Table 1 shows logistic regression analysis to identify potential factors affecting intake of different adjunctive pharmacotherapies among ACS patients in southwest Saudi Arabia.

**Table 1:** Multivariate analysis, adjusted odds ratio (aOR), and antecedent 95% confidence intervals (CI) of potential factors affecting intake of different adjunctive pharmacotherapies among ACS patients in southwest Saudi Arabia

Variable	Statin aOR (95%CI)	Beta–Blocker aOR (95%CI)	Aspirin aOR (95%CI)	Clopidogrel aOR (95%CI)	ACEI/ARBS aOR (95%CI)
Age: Below 60 vs 60+years*	0.885 (0.504–1.553)	1.419 (0.967–2.083)	0.871 (0.222–3.424)	1.448 (0.903–2.322)	0.806 (0.654–1.153)
Gender: males vs females	1.070 (0.573–1.998)	0.657 (0.420–1.027)	1.328 (0.301–5.864)	1.215 (0.739–1.99)	1.161 (0.775–1.738)
History of diabetes mellitus: yes vs no	0.799 (0.459–1.391)	1.200 (0.828–1.739)	1.327 (0.344–5.118)	1.029 (0.654–1.618)	1.496* (1.055–2.121)
History of hypertension: yes vs no	1.200 (0.675–2.133)	1.452 (0.983–2.145)	1.163 (0.278–4.862)	0.723 (0.459–1.140)	1.222 (0.850–1.757)
Smoking habit: yes vs no	1.391 (0.625–3.097)	1.065 (0.650–1.764)	2.076 (0.227–19.003)	1.058 (0.546–2.051)	1.009 (0.628–1.623)
Having UA: yes vs no	9.803* (1.312–71.42)	1.186 (0.656–2.145)	1.167 (0.126–10.831)	0.884 (0.443–1.764)	0.629 (0.358–1.107)
Having STEMI: yes vs no	8.064* (1.052–62.5)	0.884 (0.476–1.639)	0.636 (0.067–6.050)	2.026 (0.912–4.502)	1.058 (0.578–1.937)
Having NSTEMI: yes vs no	1.320 (0.695–2.987	1.329 (0697–1.983)	1.170 (0.473–1.921)	1.121 (0.196–7.841)	1.119 (0.911–2.113)

* Significant (*P*<0.05)

Patients with the diagnosis of UA have significantly 9.8 times the chance of being on statin therapy compared to patients without the diagnosis of UA (adjusted odds ratio [aOR] = 9.803, 95% confidence interval [CI] = 1.312–71.42).

Patients with the diagnosis of STEMI have significantly eight times the chance of being on statin therapy compared to patients without the diagnosis of STEMI (aOR =8.064, 95% CI = 1.052–62.5).

However, diabetic patients are more likely to be on ACEI (aOR = 1.496, 95% CI = 1.055–2.121).

### Survival and its determinants

Of all, 25 patients (4.4%) died and the rest were discharged alive. Table 2 shows logistic regression analysis to identify factors associated with survival.

**Table 2:** Multivariate analysis, adjusted odds ratio (aOR), and antecedent 95% confidence intervals (CI) of potential factors affecting survival among IHD patients in southwestern Saudi Arabia

Variable	aOR	95% CI	
		Lower	Upper
Age: below 60 vs 60+ years*	6.204	1.557	24.712
Gender: males vs females	0.560	0.180	1.748
History of diabetes mellitus: yes vs no	0.746	0.296	1.881
History of hypertension: yes vs no	0.807	0.311	2.095
Smoking habit: yes vs no	4.750	0.556	40.578
Intake of statin medications: yes vs no*	3.277	1.133	9.475
Intake of β–blockers: yes vs no	2.443	0.979	6.099
Intake of aspirin: yes vs no*	7.340	1.118	48.209
Intake of clopidogrel: yes vs no	1.965	0.684	5.646
Intake of ACE/ARBS inhibitors: yes vs no	1.779	0.717	4.414
Having unstable angina: yes vs no	3.418	0.739	15.819
Having ST-elevation MI: yes vs no	0.618	0.151	2.522
Having non–ST-elevation MI: yes vs no	0.941	0.557	4.712

* Significant (*P*<0.05)

Patients younger than 60 years of age have significantly 6.2 times the chance of survival compared with patients aged 60 years or older (aOR = 6.204, 95% CI = 1.557–24.712).

Patients receiving aspirin had significantly 7.3 times the chance of survival compared with those not receiving the drugs (aOR = 7.340, 95% CI = 1.118–48.209). Similarly, intake of statin was found to be significant in affecting the survival (aOR = 3.277, 95% CI = 1.133–9.475). On the other hand, the rest of medications, gender, and medical and family history were of no significant value in affecting survival.

## DISCUSSION AND CONCLUSION

In the current study, the uses of certain adjunctive pharmacotherapies, specifically β-blockers (69%) and ACEI (or angiotensin 2 receptor blocker) (59%) for patients admitted with ACS were not optimum and underutilized. This does not achieve a high level of care compared with what is recommended in international guidelines. These pharmacotherapies have an important role in improving survival on such patients, as documented by several studies.[7–13]

On the other hand, the uses of other adjunctive pharmacotherapies, specifically aspirin, clopidogrel, and lipid-lowering therapy (statin) (98.4, 82.6, and 89.3%, respectively) were optimal and in concordance with the international recommended guidelines.

The underutilization of β-blockers and ACEI can reflect on patient’s survival. Such underutilization is a multi-factorial problem and may stem from misperceptions regarding drug safety profile or from concerns about adverse effects. Other reasons may be related to patient’s compliance with treatment and physician’s attitude in complying with the evidence-based practice.

Such a gap in practice resulting from underutilization of adjunctive pharmacotherapies has been reported in different studies, where similar observation was noticed (in the Estonian experience underutilization of such an important adjunctive therapy was recognized) and found that only 40% of their patients were treated by combinations of β-blockers, ACEI/angiotensin 2 receptor blockers, and statin.[19]

Another study from Spain showed underutilization of adjunctive pharmacotherapies after myocardial infarction in a primary care setting, where β-blockers were prescribed in only 50.2% of cases, ACEI in only 32.5% of cases, and lipid-lowering therapy in only 52% of cases.[20]

Compared with the reported international experiences, our local Saudi practice suffers a similar problem (although to a lesser extent) of adherence to the recommended guidelines.

A positive relationship between adherence to evidence-based pharmacotherapy and survival after ACS has been reported.[21,22] Among factors that we studied in our cohort that may predict the utilization of the adjunctive pharmacotherapy, we found that the presence of diabetes predicts the use of ACEI, which reflects understanding the important role of ACEI in diabetic patients. Although the diagnosis of UA and STEMI predict the use of statin therapy, such therapy have been shown to reduce mortality when given to ACS patients.[14]

Among our cohort, we found that the uses of aspirin and statin therapy correlate with survival on discharge. This concurs with what the large trials showed using these therapies.[6,14]

This is the first study in Saudi Arabia that aimed to examine the pattern of utilization of different adjunctive pharmacotherapies among patients with the diagnosis of ACS. The study showed underutilization of β-blockers and ACEI in managing ACS patients. This study hopes to improve medical care by raising awareness of this problem. Physicians are urged use all pharmacotherapies that have been proven to be effective and safe in treating patients with ACS.
